# In Vitro Activity of a Novel Siderophore-Cephalosporin LCB10-0200 (GT-1), and LCB10-0200/Avibactam, against Carbapenem-Resistant *Escherichia coli*, *Klebsiella pneumoniae*, *Acinetobacter baumannii*, and *Pseudomonas aeruginosa* Strains at a Tertiary Hospital in Korea

**DOI:** 10.3390/ph14040370

**Published:** 2021-04-16

**Authors:** Le Phuong Nguyen, Chul Soon Park, Naina Adren Pinto, Hyunsook Lee, Hyun Soo Seo, Thao Nguyen Vu, Hung Mai, An H. T. Pham, Eris Jang, Young Lag Cho, Karrie Goglin, Kevin Nguyen, Richard White, Roshan D’Souza, Derrick E. Fouts, Dongeun Yong

**Affiliations:** 1 Department of Laboratory Medicine and Research Institute of Bacterial Resistance, Yonsei University College of Medicine, Seoul 03722, Korea; luongphekidz07@gmail.com (L.P.N.); cspark0523@yuhs.ac (C.S.P.); naina.pinto@gmail.com (N.A.P.); snoopycat78@gmail.com (H.L.); hsseo91@yuhs.ac (H.S.S.); vuthaonguyen1992@gmail.com (T.N.V.); eris.jang@gmail.com (E.J.); 2 Brain Korea 21 PLUS Project for Medical Science, Yonsei University, Seoul 03722, Korea; 3 School of Engineering and Applied Science, University of Pennsylvania, Philadelphia, PA 19104, USA; mhmaihung@gmail.com; 4 UCI School of Biological Sciences, University of California, Irvine, CA 92617, USA; phamhuynhthuyan@gmail.com; 5 Legochem Biosciences, Daejeon 34302, Korea; young@legochembio.com; 6 J. Craig Venter Institute, La Jolla, CA 92037, USA; kgoglin@jcvi.org; 7 J. Craig Venter Institute, Rockville, MD 20850, USA; kevinnguyentk@gmail.com (K.N.); richardcwhite89@gmail.com (R.W.); roshanbernard@gmail.com (R.D.); dfouts@jcvi.org (D.E.F.)

**Keywords:** carbapenem resistance, LCB10-0200 (GT-1), LCB10-0200/Avibactam, siderophore-antibiotic conjugate

## Abstract

The siderophore–antibiotic conjugate LCB10-0200 (a.k.a. GT-1) has been developed to combat multidrug-resistant Gram-negative bacteria. In this study, the in vitro activity of LCB10-0200 and LCB10-0200/avibactam (AVI) has been investigated against carbapenem-resistant *Escherichia coli*, *Klebsiella pneumoniae*, *Acinetobacter baumannii*, and *Pseudomonas aeruginosa*. Minimal inhibitory concentrations (MICs) of LCB10-0200, LCB10-0200/AVI, aztreonam, aztreonam/AVI, ceftazidime, ceftazidime/AVI, and meropenem were measured using the agar dilution method. Whole genome sequencing was performed using Illumina and the resistome was analyzed. LCB10-0200 displayed stronger activity than the comparator drugs in meropenem-resistant *E. coli* and *K. pneumoniae*, and the addition of AVI enhanced the LCB10-0200 activity to MIC ≤ 0.12 mg/L for 90.5% of isolates. In contrast, whereas LCB10-0200 alone showed potent activity against meropenem-resistant *A. baumannii* and *P. aeruginosa* at MIC ≤ 4 mg/L for 84.3% of isolates, the combination with AVI did not improve its activity. LCB10-0200/AVI was active against CTX-M-, SHV-, CMY-, and KPC- producing *E. coli* and *K. pneumoniae*, while LCB10-0200 alone was active against ADC-, OXA-, and VIM- producing *A. baumannii* and *P. aeruginosa*. Both LCB10-0200 and LCB10-0200/AVI displayed low activity against IMP- and NDM- producing strains. LCB10-0200 alone exhibited strong activity against selected strains. The addition of AVI significantly increased LCB10-0200 activity against carbapenem-resistant *E. coli*, *K. pneumoniae*.

## 1. Introduction

Carbapenem-resistant *Escherichia coli*, *Klebsiella pneumoniae*, *Acinetobacter baumannii*, and *Pseudomonas aeruginosa* have been recognized as being of “critical priority” to the research and development of new antibiotics according to the World Health Organization [1]. Even though colistin has been used as a last resort treatment of carbapenem-resistant bacteria, the emergence of its resistance has been reported worldwide [2,3,4]. A similar plight has been observed with ceftazidime/avibactam (CAZ-AVI), an antibiotic approved by the US Food and Drug Administration (FDA) in 2015 [5]. Recently, the emergence of NDM-, KPC- and/or MCR-1 co-producing *E. coli* and *K. pneumoniae* strains have been discovered [6,7,8,9]. These strains are resistant to both carbapenems and colistin, limiting treatment choices in clinical settings. Therefore, the need for developing new antibiotics that are active against carbapenem-resistant strains is highly critical and urgent. Recently, LegoChem Biosciences (Daejeon, Korea) and Geom Therapeutics (San Francisco, CA, USA) have developed the novel siderophore-cephalosporin LCB10-0200 (a.k.a. GT-1), which increases the antibiotics’ influx into bacteria via the siderophore uptake system, and could potentially treat carbapenem-resistant bacterial infections [10].

In a previous study, our group evaluated the in vitro activity of LCB10-0200 against panels of well-characterised *E. coli*, *K. pneumoniae*, and *Acinetobacter* spp. strains showing diverse antibiograms [11,12,13]. Panel strains of these three species were classified into different groups, including Non-Extended Spectrum β-lactamase (Non-ESBL), ESBL-, ESBL-AmpC-, AmpC-carbapenemase- and ESBL-carbapenemase-producing strains. LCB10-0200 exhibited MICs ≤2 mg/L against multi-drug resistant isolates, including ESBL- (CTX-M-14, CTX-M-15, SHV-12, SHV-83), AmpC- (CMY-2, ADC-22, ADC-25, ADC-30, ADC-77) and carbapenemase- (KPC-2, IMP-1) producing *E. coli* and *K. pneumoniae* and OXA- (OXA-23, OXA-48, OXA-66, OXA-82, OXA-120, OXA-213, OXA-421, OXA-499) producing *Acinetobacter* spp. In the current study, we focused on the investigation of LCB10-0200′s activity against carbapenem-resistant *E. coli*, *K. pneumoniae*, *A. baumannii*, and *P. aeruginosa* and compared its activity with aztreonam (ATM), ceftazidime (CAZ), and meropenem (MEM). Moreover, avibactam (AVI), a second generation β-lactamase inhibitor, was also included in this study. AVI covalently binds and inhibits Ambler class A, class C, and some class D β-lactamases [14,15,16]. As a result, AVI can reverse the activity of CAZ in CAZ-resistant strains [17,18]. The combination of AVI and CAZ was approved by the US FDA as treatment for complicated intra-abdominal infections (cIAI), complicated urinary tract infections, hospital acquired bacterial pneumonia, and ventilator-associated bacterial pneumonia [19]. In addition, the combination of AVI with ATM also demonstrated good activity against Ambler class A/C and class B β-lactamase-coproducing strains [20]. This combination was studied in a phase IIa clinical trial for the treatment of cIAI [21]. Until now, the in vitro activity of a siderophore-cephalosporin and AVI combination has not been well studied. For that reason, this study also investigated the synergistic activity of AVI and LCB10-0200 in comparison with CAZ-AVI and ATM-AVI in vitro. The higher MICs of LCB10-0200 and LCB10-0200/AVI were correlated with the corresponding resistome profiles to explain the underlying resistance mechanisms.

## 2. Results

**Figure 1:**
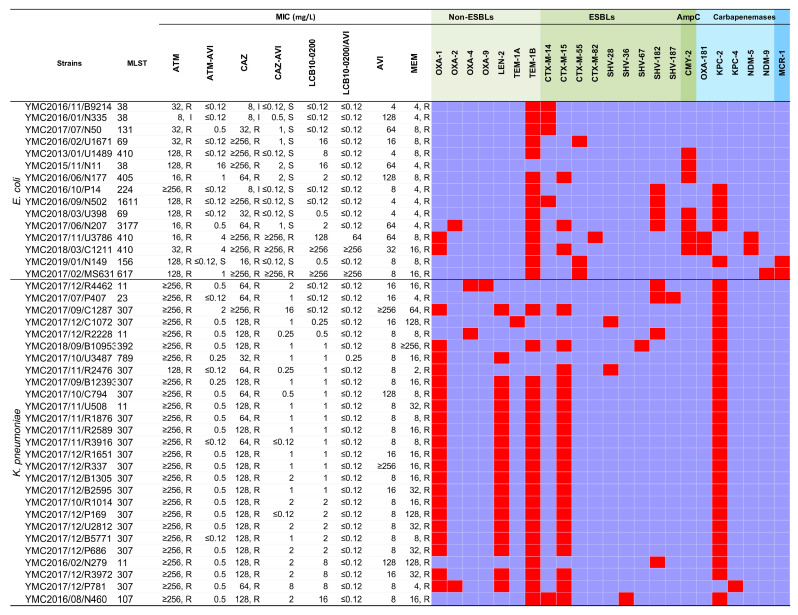
Antibiotic susceptibility and resistome of the carbapenem-resistant *E. coli* and *K. pneumoniae* strains.Abbreviation: ATM, aztreonam; AVI: Avibactam; ATM-AVI: aztreonam/avibactam; CAZ, ceftazidime; CAZ-AVI, ceftazidime/avibactam; MEM, meropenem; ESBL, Extended-spectrum β-lactamase. Antibiotic susceptibility testing was performed using agar dilution method. Data were interpreted using the Clinical and Laboratory Standards Institute guidelines M100 28th ed. The orchid and red color indicate the absence and presence of antimicrobial resistance genes, respectively. The very light green, light green, green, light blue indidate β-lactamses belonging to Non-ESBLs, ESBLs, AmpC, and carbapenemases. The blue indicates colisin-resistant enzyme MCR-1.

A total of ninety-three clinical isolates were collected in this study, consisting of 16% *E. coli*, 29% *K. pneumoniae*, 27% *A. baumannii*, and 28% *P. aeruginosa*, which were further divided into two subsets (i.e., fermenting Gram-negative bacilli (*E. coli* and *K. pneumoniae*) and non-fermenting Gram-negative bacilli (*A. baumannii*, and *P. aeruginosa*) (Table 1). These bacteria were isolated from various clinical samples, such as stool, blood, pus, urine, respiratory fluid and cerebrospinal fluid. All isolates were resistant to MEM with the exception of *K. pneumoniae* YMC2017/11/R2476 (MEM MIC = 2 mg/L, intermediate). All of the strains harboured more than one β-lactamase gene, except *E. coli* YMC2015/11/N11 (Figure 1). The MICs of ATM-AVI, LCB10-0200, and LCB10-0200/AVI for *E. coli* ATCC 25922 were ≤0.12 mg/L, whereas for *P. aeruginosa* ATCC 27853 they were 4, ≤0.12–0.5, and ≤0.12–0.5 mg/L, respectively. The MIC of ATM, CAZ, and CAZ-AVI for the quality control strains was within the CLSI recommended ranges [22].

### 2.1. LCB10-0200, and LCB10-0200/AVI Displayed Potent Activity against Carbapenem-Resistant E. coli and K. pneumoniae Strains

**Figure 2:**
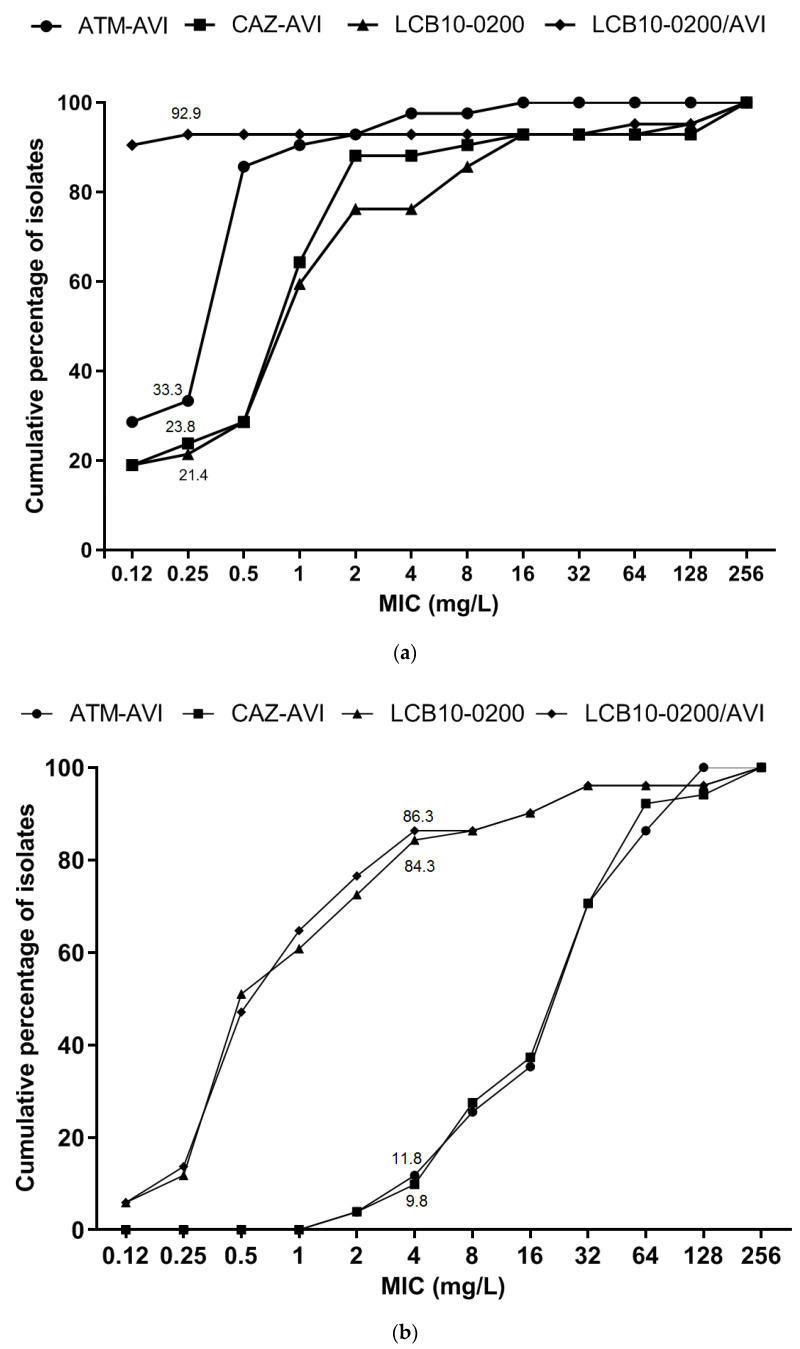
Distribution and cumulative percentages of tested isolates for (**a**) carbapenem-resistant *E. coli* and *K. pneumoniae* (*n* = 42), and (**b**) *A. baumannii* and *P. aeruginosa* (*n* = 51).

Different types of β-lactamases were identified in the tested *E. coli* and *K. pneumoniae* strains from genomic data, including non-ESBLs (OXA-1, OXA-2, OXA-4, OXA-9, LEN-2, TEM-1A, TEM-1B), ESBLs (CTX-M-14, CTX-M-15, CTX-M-55, CTX-M-82, SHV-28, SHV-36, SHV-67, SHV-182, SHV-187), AmpC β-lactamase (CMY-2), carbapenemases (KPC-2, KPC-4, NDM-5, NDM-9), and MCR-1 (Figure 1). LCB10-0200 demonstrated a wide MIC range of ≤0.12–≥256 mg/L, a MIC_50_ of 1 mg/L, and a MIC_90_ of 16 mg/L. LCB10-0200 MIC_50_ was 256-fold and 128-fold lower than ATM and CAZ, respectively. LCB10-0200 MIC_90_ was at least 16-fold lower than ATM and CAZ. In addition, LCB10-0200 in combination with AVI reduced the LCB10-0200 MIC_90_ by at least seven doubling dilutions from 16 to ≤0.12 mg/L (Table 1). LCB10-0200/AVI MIC_90_ was at least eight-fold and 64-fold lower than that of ATM-AVI and CAZ-AVI, respectively. Importantly, LCB10-0200 MIC_50_ and LCB10-0200 MIC_90_ were eight-fold lower than MEM MIC_50_ and MEM MIC_90_. In the case of LCB10-0200/AVI form, there was a 512-fold decrease in LCB10-0200/AVI MIC_90_ compared to that of MEM. ATM-AVI, CAZ-AVI, LCB10-0200, and LCB10-0200/AVI MICs were ≤0.25 mg/L for 33.3%, 23.8%, 21.4%, and 92.9% of meropenem-resistant Enterobacteriaceae isolates, respectively (Figure 2a). Among the KPC- producing strains, LCB10-0200 MIC_90_ was at 8 mg/L and the addition of AVI shifted the MIC_90_ value to ≤0.12 mg/L. As a result, LCB10-0200/AVI MIC_90_ resulted in at least 16-fold lower MIC than CAZ-AVI MIC_90_ in KPC-producing strains (Table 1). LCB10-0200 MIC was high (≥128 mg/L) in the NDM-5, CMY-2 co-producing *E. coli* YMC/2017/11/U3786, YMC2018/03/C1211, and in NDM-9, MCR-1 co-producing *E. coli* YMC/2017/02/MS631 (Figure 1). These findings suggested that LCB10-0200 is inactive against NDM- producing strains.

### 2.2. In Vitro Activity of LCB10-0200, and LCB10-0200/AVI against A. baumannii, and P. aeruginosa Strains

**Figure 3:**
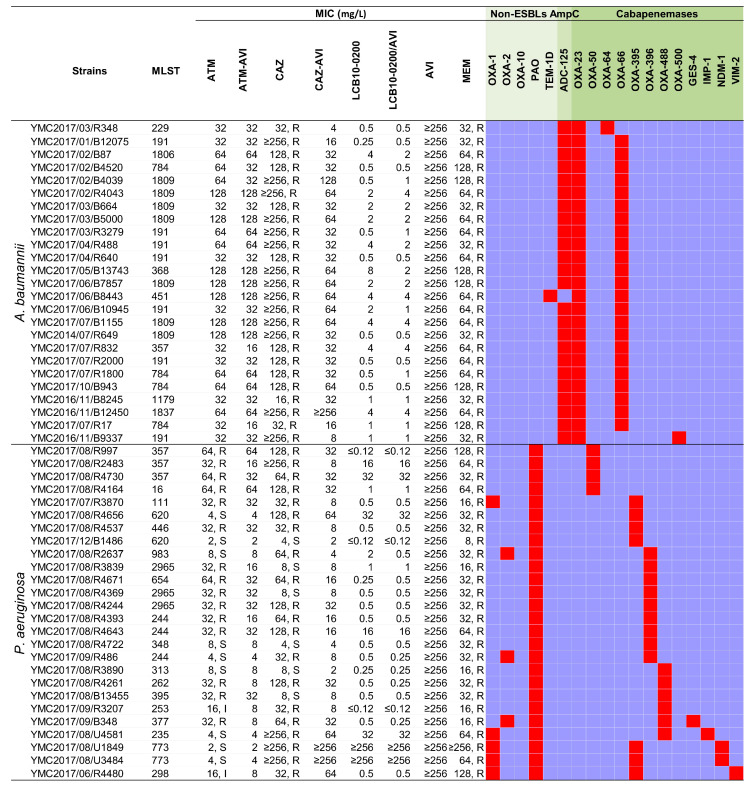
Antibiotic susceptibility and resistome of the carbapenem-resistant *A. baumannii* and *P. aeruginosa* strains.Abbreviation: ATM, aztreonam; ATM-AVI: aztreonam/avibactam; CAZ, ceftazidime; CAZ-AVI, ceftazidime/avibactam; MEM, meropenem; ESBL, Extended-spectrum β-lactamase. Antibiotic susceptibility testing was performed using agar dilution method. Data interpretation was carried out using the CLSI guidelines M100 28th ed. The orchid and red color indicate the absence and presence of antimicrobial resistance genes, respectively. The very light green, light green, green indidate β-lactamses belonging to Non-ESBLs, AmpC, and carbapenemases, respectively.

A variety of β-lactamases were also identified from the genomes of the collected *A. baumannii* and *P. aeruginosa* strains, including non-ESBL’s (OXA-1, OXA-2, OXA-10, PAO, TEM-1D, TEM-187), AmpC (ADC-25), and carbapenemases (OXA-23, OXA-50, OXA-64, OXA-66, OXA-395, OXA-396, OXA-488, OXA-500, GES-4, IMP-1, NDM-1, VIM-2) (Figure 3). All of the *A. baumannii* strains were co-producing at least three β-lactamases and eight out of twenty-six *P. aeruginosa* strains harbored at least three β-lactamase genes. The LCB10-0200 MIC_50_ was at 0.5 mg/L, which was 64-fold, and 256-fold lower than ATM, and CAZ, respectively. However, the LCB10-0200 MIC_90_ was 16 mg/L, which was 8-fold, and 16-fold lower than ATM, and CAZ, respectively. MIC_50_ and MIC_90_ of LCB10-0200 were 128-fold and 8-fold lower than those in MEM. In addition, LCB10-0200 alone displayed more potent activity compared to CAZ-AVI and ATM-AVI, of which, LCB10-0200 MIC_90_ was 8-fold, and 4-fold lower than CAZ-AVI and ATM-AVI, respectively. However, the addition of AVI did not change the LCB10-0200 MIC_90_, which suggested that AVI did not efficiently enhance the LCB10-0200 activity in meropenem-resistant non-fermenting Gram-negative bacilli. There was a two-fold increase in MIC when LCB10-0200 was combined with AVI in the *A. baumannii* strains YMC2017/01/B12075, YMC2017/02/B4039, YMC2017/02/R4043, YMC2017/03/R3279, and YMC2017/07/R1800. This caused an increase in LCB10-0200/AVI MIC_50_ by 1 mg/L. There was no change in the LCB10-0200/AVI MIC_90_ of 16 mg/L, although LCB10-0200 MIC reduced two to four-fold in combination with AVI in *A. baumannii* strains YMC2017/02/B87, YMC2017/04/R488, YMC2017/05/B13743, and YMC2017/06/B10945. ATM-AVI, CAZ-AVI, LCB10-0200, and LCB10-0200/AVI MICs were ≤4 mg/L for 11.8%, 9.8%, 84.3%, and 86.3% of meropenem-resistant non-fermenting Gram-negative bacteria (Figure 2b). LCB10-0200 displayed a MIC range of ≤0.12–32 mg/L, a MIC_50_ of 0.5 mg/L, and a MIC_90_ of 4 mg/L against OXA-producing strains. Similarly, against metallo-carbapenemase producing strains, LCB10-0200 MICs were at 0.5 mg/L in the GES-4 and VIM-2 producing strains (YMC2017/09/B348, YMC2017/06/R4480). However, relatively high MIC of 32 and 64 mg/L were observed in the IMP-1 producing strain (YMC2017/08/U4581). Furthermore, there was an extremely high LCB10-0200 MIC of ≥256 mg/L in NDM-1 producing *P. aeruginosa* YMC2017/08/U1849 and YMC2017/08/U3484.

## 3. Discussion

Antimicrobial resistance has been recognized as a global public health issue. Currently, the annual number of deaths caused by bacterial infection is approximately 700,000 in the entire world. This number is predicted to be around 10,000,000 deaths with the cost of around 100 trillion dollars by 2050 [23]. According to USA CDC, the number of new cases per year increased by approximately 29% from 2 million in 2013 [24] to 2.8 million in 2019 [25]. Furthermore, the number of deaths increased by 20% from 28,000 in 2013 to 35,000 in 2019. However, this status may be worsened due to the emergence of the coronavirus disease 2019 (COVID-19) pandemic, which is caused by severe acute respiratory syndrome coronavirus 2 (SARS-CoV-2). Indeed, the infection caused by viruses creates opportunistic chances for co-infection with bacteria. Since the beginning of COVID-19, there have been extensive studies and systematic reviews in terms of the co-infection between SARS-CoV-2 and secondary bacterial infection around the world. The ratio of bacterial superinfection in COVID-19 patients ranged from 3.2% to 15% in a UK secondary-care setting and Wuhan hospitals [26,27]. According to a cohort study conducted in a hospital in Barcelona, Spain, 4.7% of COVID-19 patients were co-infected with *P. aeruginosa* or *E. coli* with an average time from admission to bacterial infection diagnosis of 10.6 days [28]. Another systematic analysis of postmortem studies conducted by Clancy et al. identified that 8% of patients were infected with SARS-CoV-2 and bacteria and 24% of patients who died by SARS-CoV-2 were possibly co-infected with bacteria [29]. The highest ratio of secondary bacterial infection in this study was *A. baumannii*, followed by *Staphylococcus aureus*, *P. aeruginosa*, and *K. pneumoniae* [29]. Moreover, it was reported that 71% of patients admitted by SARS-CoV-2 in hospitals in China were treated by broad-spectrum antibiotics without the confirmation of secondary bacterial infection to save patient lives and to reduce the additional complications [30]. Consequently, this fact may have driven the antibiotic resistance rate in the COVID-19 hotspots. On the other hand, there are some good signs that can affect the worldwide antibiotic resistance climate. Firstly, the reduction of travelers all over the world during COVID-19 pandemic can subsequently reduce the spread of different types of antimicrobial-resistant bacteria from regions to regions. Secondly, stringent hand hygiene, self-quarantine, and social distancing in the community and health facilities can decrease not only the spread of SARS-CoV-2 but also the cross-infection of antibiotic- resistant bacteria [31]. However, the concerns about the higher ratios of antibiotic resistance in the COVID-19 aftermath should be considered and research & development of new antibiotics should be conducted in more efficient ways in parallel with enhanced antimicrobial stewardship programs. Even though antimicrobial resistance is one of the greatest threats in the mid-twenty-first century, financial investment in antimicrobial development has reduced in recent years due to the low rate of success and revenue as compared to its high investment cost [32]. Payne et al. indicated that approximately 3.5% of candidates from high throughput screening can reach to phase I of clinical trials. According to the European observatory on health systems and policies, the success rates for an antibiotic candidate in phase I → II, II → III, and III → IV are 33%, 59.3%, and 75.8%, respectively, and it takes around 13–21 years for a candidate to be available on market [33]. Once a new antibiotic is approved, it is used as a last resort and therefore limits the profitability. Another difficulty in the development of new antibiotics is the limitation of traditional drug discovery platforms, which usually results in quite similar drug structures or previously identified targets [34,35]. Low permeability on the bacterial membranes, especially in Gram-negative bacteria is also the cause of the failure in the early stage of antibiotic development [33]. Other factors such as variations in drug targets, drug hydrolyses, overexpression of efflux pumps, and porin losses are also the barriers in the later stages of novel antibiotic development [33]. However, some strategies have been applied to tackle these challenges. Firstly, various non-profit and government-based programs such as European Gram-negative antibacterial engine (ENABLE), combating bacterial resistance in Europe (COMBACTE), US Biomedical Advanced Research and Development Authority (BARDA), Global Antibiotic Research and Development Partnership (GARDP), and Combating Antibiotic-Resistant Bacteria Biopharmaceutical Accelerator (CARB-X) have been implemented to foster novel antibiotic development [36,37,38,39,40]. Secondly, novel approaches including inhaled delivery and liposomal delivery have been developed to increase antibiotic concentration in lung infection and to overcome the low drug permeability [41,42]. One way to improve the drug influx into the bacterial membrane is the conjugation between antibiotic and siderophore, a.k.a “Trojan horse” strategy, which was applied in the development of LCB10-0200 [11]. Importantly, recent advances in bioinformatics, machine learning, and deep learning have been applied in prediction of antimicrobial molecules [43,44]. Recently, Stokes et al. applied different neural network algorithms including Chemprop and ensembling to learn and predict antimicrobial properties from theirs chemical structures. A set of 2335 molecules from a FDA-approved drug library and a modest natural product library were used as a training set for growth inhibition against *E. coli* BW25113. The trained model was then applied to predict antimicrobial molecules from a set of 6111 molecules from the Drug Repurposing Hub and identified a broad-spectrum antimicrobial molecule, Hacilin [44]. Importantly, the structure of Haicilin is structurally divergent from current antibiotics [44]. This approach can overcome one of the shortcomings of conventional drug screening, in which the candidate structures are quite similar to known antibiotics. Also, this approach can reduce time and cost for drug library screening and development.

To cope with the predicted and potential scenarios of antibiotic resistance, our group explored the in vitro activity of LCB10-0200 alone and in combination with AVI against multiple carbapenem-resistant Gram-negative clinical isolates with various carbapenem resistance determinants. The predominant resistance mechanisms observed in this study belonged to KPC- and OXA- producing strains. Against KPC- producing strains, LCB10-0200 had a high activity with the MIC range of ≤0.12–16 mg/L, and a MIC_50_ of 1 mg/L. The MIC_90_ in KPC- producing strains was also 8 mg/L, being similar with the previous report [11]. LCB10-0200 activity was significantly enhanced in combination with AVI, (i.e., LCB10-0200/AVI MIC_90_ was at least 16-fold lower than CAZ-AVI MIC_90_). Of note, CAZ-AVI resistant *K. pneumoniae* strains have increased prevalence in many parts of the world in recent years due to the spread of a mutation in the omega loop of KPC-2 and KPC-3 [45,46,47,48]. This has prompted an urgent need to develop new antimicrobial agents against these resistant strains. Even though, there was no CAZ-AVI resistant strain detected in this study, the potent activity of LCB10-0200/AVI against KPC-producing strains has shown promising results, and further studies need to be carried out to determine the activity of LCB10-0200/AVI against CAZ-AVI resistant KPC-producing *K. pneumoniae*.

It was well-described in the literature that one of the most common carbapenem resistance mechanisms in *A. baumannii* and *P. aeruginosa* is possession of OXA-23 and other OXA-type carbapenemases [49,50,51,52]. In our previous study, the in vitro activity of LCB10-0200 against the classified panel strains showing diverse carbapenem susceptibility was measured [11]. For example, in *A. baumannii* panel strains, there were 11 isolates including narrow-spectrum oxacillinase (one isolate), ESBL-AmpC beta-lactamase (three isolates), ESBL-AmpC beta-lactamase (two strains), ESBL-AmpC-carbapenemase-producing strains (five strains). However, in the current study, we measured LCB10-0200 activity on an additional 25 carbapenem-resistant *A. baumannii* strains. The data revealed the lowest MIC_90_ of LCB10-0200 among comparators against the additional OXA-type carbapenemases, which have not been previously reported, including OXA-50, OXA-64, OXA-66, OXA-395, OXA-396, OXA-488, OXA-500.

In addition, carbapenem-resistant *E. coli*, *K. pneumoniae*, and *P. aeruginosa* strains were selected. Of interest, LCB10-0200 was active against GES-4, or VIM-2 producing strains, but inactive against NDM-1, NDM-5, and NDM-9 producing strains. Addition of AVI did not enhance the activity of LCB10-0200. This was consistent with the fact that AVI has limited, or no activity against metallo-β-lactamase-producing strains [53]. The LCB10-0200 MIC against IMP-1 producing *P. aeruginosa* strain (YMC2017/08/U4581) was 32mg/L, which was 64-fold higher than the LCB10-0200 MIC of IMP-1 producing *K. pneumoniae* YMC2012/08/C631 in the previous study (0.5 mg/L) [11]. The discrepancy may be due to the reduced background of β-lactamases in the *K. pneumoniae* YMC2012/08/C631. Studies using more IMP-producing strains should be performed to get better insights.

## 4. Materials and Methods

### 4.1. Specimen Collection and Antibiotics

A total of 93 non-duplicate clinical isolates, including 15 *E. coli*, 27 *K. pneumoniae*, 25 *A. baumannii*, and 26 *P. aeruginosa* strains were collected during 2015–2018 in a University- affiliated hospital in Korea. Species identification was confirmed using matrix-assisted laser desorption ionization-time of flight mass spectrometry (MALDI-TOF MS) (ASTA, Suwon, Korea) according to the manufacturer instructions. In brief, the single bacterial colony was smeared on the target plate, followed by 1–2 μL of 70% formic acid (Sigma, St. Louis, MO, USA). After 3–5 min for air-drying, 1–2 μL of matrix solution (α-cyano-4-hydroxycinnamic acid was overlaid on the same spot followed by an additional air-dry step. Finally, the peptide profile was obtained using ASTA MicroIDSys with the coreDB v1.26 and the mass spectra ranging from 2000 to 20,000 daltons. *E. coli* protein (YbdYbiotech, Seoul, Korea) was used as calibrator. Antibiotics used in this study include ATM (Dong-A Biotech Co., Seoul, Korea), CAZ (CJ Health Care, Seoul, Korea), and MEM (Yuhan Co., Seoul, Korea). AVI was kindly provided by LegoChem Biosciences. LCB10-0200 was manufactured by LegoChem Biosciences.

### 4.2. Susceptibility Tests and MIC Determinations

Minimum inhibitory concentrations (MICs) were determined using the agar dilution method and interpreted according to the CLSI guidelines [22,54]. Antibiotic concentrations used ranged from 0.12 mg/L to 256 mg/L. MIC interpretation for LCB10-0200, LCB10-0200/AVI, and ATM-AVI MIC was not available at the time of this study. A previous study reported that LCB10-0200 MICs against bacteria grown on Muller Hinton medium did not vary significantly as compared to iron-depleted Muller Hinton medium [55]. Therefore, the in vitro activity of LCB10-0200 in iron-depleted medium was not investigated in this study.

### 4.3. DNA Extraction and Whole Genome Sequencing

Bacterial genomic DNA (gDNA) extraction was performed using the Wizard genomic DNA purification kit (Promega, WI, USA). The quantity and quality of gDNA was measured using a NanoDrop spectrophotometer (ND-2000 Thermo Fisher Scientific, Waltham, MA, USA) and agarose gel-electrophoresis. Whole genome sequencing was performed at different centers (Supplementary Table S1). *E. coli* strains and some *K. pneumoniae* strains were sequenced at Korea Research Institute of Bioscience & Biotechnology (KRIBB, Daejeon, Korea) and Life’s Art of Science (LAS, Gimpo, Korea). The libraries were prepared using TruSeq Nano DNA Library Preparation Kit and sequencing was performed on Illumina MiSeq platform (Illumina, CA, USA) using MiSeq reagent Kit v3 (600 cycles—2 × 300). Sequencing of select *K. pneumoniae*, *A. baumannii*, and *P. aeruginosa* isolates was performed at the J. Craig Venter Institute (JCVI, CA, USA) where the libraries were prepared using the Nextra XT library kit and sequencing was performed on the Illumina NextSeq 500 instrument using the NextSeq 500 High Output Kit (300 cycles—2 × 150).

### 4.4. Sequence Assembly, Genome Annotation, Multi-Locus Sequence Typing (MLST) and Resistome Analysis

The strains sequenced at KRIBB and LAS were trimmed using Trimmomatic v0.39 with default settings followed by assembly with SPAdes v3.13 using careful mode [56,57]. The strains sequenced at JCVI were trimmed using Trimmomatic v0.32 (settings: ILLUMINACLIP, illumna_adapters.fa:2: 30:10; LEADING, 3; TRAILING, 3; SLIDING WINDOW, 4:24; and MINLEN, 60) and assembled using SPAdes v 3.1.1, and can be obtained under NCBI BioProject PRJNA508406. Annotation was performed using the RAST server [58]. The resistome profiles were investigated using Resfinder v.3.1 [59] and verified using NCBI BLAST [60]. Geneious pro 8.1.9 (https://www.geneious.com (accessed on 14 April 2021)) was used for genomic analysis. Bacterial sequence typing was conducted using MLST tool 1.8 [61].

## 5. Conclusions

LCB10-0200 displayed stronger activity than its comparators against meropenem-resistant *E. coli* and *K. pneumoniae*. The addition of AVI enhanced the LCB10-0200 activity to MIC ≤0.12 mg/L for 90.5% of the isolates. In contrast, LCB10-0200 alone showed potent activity against meropenem-resistant *A. baumannii*, and *P. aeruginosa* at MIC ≤4 mg/L for 84.3% isolates, and the combination with AVI did not improve its activity significantly. LCB10-0200/AVI was very active against CTX-M-, SHV-, CMY-, and KPC- producing *E. coli* and *K. pneumoniae*, while LCB10-0200 alone was active against ADC-25, OXA-, and VIM- producing *A. baumannii* and *P. aeruginosa*. Both LCB10-0200 and LCB10-0200/AVI displayed low activity against GES-, and NDM- producing strains. LCB10-0200 and LCB10-0200/AVI can be a potential treatment for patients infected by carbapenem-resistant strains carrying CTX-M-, SHV-, CMY-, KPC-, ADC-25, OXA-, and VIM- β-lactamases.

## Figures and Tables

**Table 1:** MIC_50_, MIC_90_, MIC ranges, and interpretations of LCB10-0200, LCB10-0200/AVI, ATM, ATM-AVI, CAZ, CAZ-AVI, AVI against carbapenem-resistant *E. coli*, *K. pneumoniae*, *A. baumannii*, and *P. aeruginosa*.

Species (No. of Isolates, Percentages of Isolates)	MIC Data (mg/L)			MIC Interpretation (%)		
	MIC_50_	MIC_90_	Range	Susceptible	Intermediate	Resistant
All isolates (93, 100%)						
ATM	64	≥256	2–≥256	NA	NA	NA
ATM-AVI	4	64	≤0.12–128	NA	NA	NA
CAZ	128	≥256	2–≥256	NA	NA	NA
CAZ-AVI	8	64	≤0.12–≥256	NA	NA	NA
LCB10-0200	1	16	≤0.12–≥256	NA	NA	NA
LCB10-0200/AVI	0.5	16	≤0.12–≥256	NA	NA	NA
AVI	≥256	≥256	4–≥256	NA	NA	NA
MEM	32	128	2–≥256	NA	NA	NA
Fermenting gram-negative bacilli: *E. coli & K. pneumoniae* isolates (42)						
ATM	≥256	≥256	8–≥256	0	2.4	97.6
ATM-AVI	0.5	1	≤0.12–16	NA	NA	NA
CAZ	128	≥256	2–≥256	2.4	4.8	92.9
CAZ-AVI	1	8	≤0.12–≥256	NA	NA	NA
LCB10-0200	1	16	≤0.12–≥256	NA	NA	NA
LCB10-0200/AVI	≤0.12	≤0.12	≤0.12–≥256	NA	NA	NA
AVI	8	128	4–≥256	NA	NA	NA
MEM	8	64	2–≥256	0	2.4	97.6
Non-fermenting gram-negative bacilli: *A. baumannii & P. aeruginosa* (51)						
ATM	32	128	2–128	NA	NA	NA
ATM-AVI	32	128	2–128	NA	NA	NA
CAZ	128	≥256	4–≥256	11.6	1.9	88.5
CAZ-AVI	32	64	2–≥256	NA	NA	NA
LCB10-0200	0.5	16	0.12–≥256	NA	NA	NA
LCB10-0200/AVI	1	16	0.12–≥256	NA	NA	NA
AVI	≥256	≥256	≥256	NA	NA	NA
MEM	64	128	8–≥256	0	0	100
*E. coli* isolates (15, 16%)						
ATM	32	128	8–≥256	0	6.7	93.3
ATM-AVI	0.12	4	0.12–16	NA	NA	NA
CAZ	64	≥256	2–≥256	0	20	80
CAZ-AVI	1	≥256	≤0.12–≥256	80	0	20
LCB10-0200	2	≥256	≤0.12–≥256	NA	NA	NA
LCB10-0200/AVI	≤0.12	≥256	≤0.12–≥256	NA	NA	NA
AVI	16	128	4–128	NA	NA	NA
MEM	8	16	4–16	0	0	100
*K. pneumoniae* isolates (27, 29%)						
ATM	≥256	≥256	128–≥256	0	0	100
ATM-AVI	0.5	0.5	0.12–2	NA	NA	NA
CAZ	128	128	32–≥256	0	0	100
CAZ-AVI	1	2	≤0.12–16	96.3	NA	3.7
LCB10-0200	1	8	≤0.12–16	NA	NA	NA
LCB10-0200/AVI	0.12	0.12	≤0.12–0.25	NA	NA	NA
AVI	8	128	8–≥256	NA	NA	NA
MEM	8	128	2–≥256	0	3.7	96.3
KPC-producing *E. coli & K. pneumoniae* isolates (32)						
ATM	≥256	≥256	16–≥256	0	0	100
ATM-AVI	0.5	0.5	≤0.12–2	NA	NA	NA
CAZ	128	128	8–≥256	0	3.1	96.9
CAZ-AVI	1	2	≤0.12–16	96.9	3.1	0
LCB10-0200	1	8	≤0.12–16	NA	NA	NA
LCB10-0200/AVI	≤0.12	≤0.12	≤0.12–0.25	NA	NA	NA
AVI	8	128	4–≥256	NA	NA	NA
MEM	16	128	2–≥256	0	3.1	96.9
*A. baumannii* isolates (25, 27%)						
ATM	64	128	32–128	NA	NA	NA
ATM-AVI	64	128	16–128	NA	NA	NA
CAZ	≥256	≥256	16–≥256	0	4	96
CAZ-AVI	32	64	4–≥256	NA	NA	94
LCB10-0200	1	4	0.25–8	NA	NA	NA
LCB10-0200/AVI	1	4	0.5–4	NA	NA	NA
AVI	≥256	≥256	≥256	NA	NA	NA
MEM	64	128	32–128	0	0	100
*P. aeruginosa* isolates (26, 28%)						
ATM	32	64	2–64	34.6	7.7	57.7
ATM-AVI	8	32	2–64	NA	NA	NA
CAZ	64	≥256	4–≥256	23.1	NA	76.9
CAZ-AVI	16	64	2–≥256	46.2	NA	53.8
LCB10-0200	0.5	32	0.12–≥256	NA	NA	NA
LCB10-0200/AVI	0.5	32	0.12–≥256	NA	NA	NA
AVI	≥256	≥256	≥256	NA	NA	NA
MEM	32	64	8–≥256	0	0	100
OXA-type producing *A. baumannii* & *P. aeruginosa* (46)						
ATM	32	128	2–128	NA	NA	NA
ATM-AVI	32	128	2–128	NA	NA	NA
CAZ	128	256	4–≥256	13	2.2	84.8
CAZ-AVI	32	64	2–≥256	30.4	10.9	58.7
LCB10-0200	0.5	4	≤0.12–32	NA	NA	NA
LCB10-0200/AVI	1	4	≤0.12–32	NA	NA	NA
AVI	≥256	≥256	≥256	NA	NA	NA
MEM	32	128	8–128	0	0	100

Abbreviation: ATM, aztreonam; AVI: avibactam; ATM-AVI: aztreonam/avibactam; CAZ, ceftazidime; CAZ-AVI, ceftazidime/avibactam; MEM, meropenem; NA, Not available.

## Data Availability

Data sharing not applicable.

## Supplementary Materials

The following are available online at https://www.mdpi.com/article/10.3390/ph14040370/s1, Table S1: GenBank Accession numbers and the sequencing sites for all the tested strains.
