# Supplementary material for: In Vitro Activity of a Novel Siderophore-Cephalosporin LCB10-0200 (GT-1), and LCB10-0200/Avibactam, against Carbapenem-Resistant Escherichia coli, Klebsiella pneumoniae, Acinetobacter baumannii, and Pseudomonas aeruginosa Strains at a Tertiary Hospital in Korea
Source: Pharmaceuticals (Basel). 2021 Apr 16;14(4):370. doi: 10.3390/ph14040370 (PMC8072773; doi:10.3390/ph14040370)
Supplement: Supplementary file 1 [file pharmaceuticals-14-00370-s001.zip › Table S1.pdf]

Table S1. GenBank Accession numbers and the sequencing sites for all the tested strains

Species	Strains	Accession numbers	Sample numbers	Sequencing sites
	YMC2016/11/B9214	WAJH00000000	SAMN12855557	KRIBB
	YMC2016/01/N335	WAIH00000000	SAMN12854952	KRIBB
	YMC2017/07/N50	WAJC00000000	SAMN12855525	KRIBB
	YMC2016/02/U1671	WAIK00000000	SAMN12854957	KRIBB
	YMC2016/10/P14	WAJO00000000	SAMN12856460	KRIBB
	YMC2016/09/N502	WAJL00000000	SAMN12856441	KRIBB
	YMC2018/03/U398	WAIR00000000	SAMN12854981	KRIBB
E. coli	YMC2016/06/N177	WAJF00000000	SAMN12855554	KRIBB
	YMC2017/06/N207	WAJB00000000	SAMN12855383	KRIBB
	YMC2013/01/U1489	WAAA00000000	SAMN12826519	KRIBB
	YMC2015/11/N11	WAJK00000000	SAMN12856440	KRIBB
	YMC2017/11/U3786	WAJN00000000	SAMN12856459	KRIBB
	YMC2018/03/C1211	WAIY00000000	SAMN12855204	KRIBB
	YMC2019/01/N149	JAEEGD000000000	SAMN16992981	LAS
	YMC2017/02/MS631	SBHK00000000	SAMN10715997	KRIBB
	YMC2017/12/R4462	RWYB00000000	SAMN10523669	JCVI
	YMC2017/07/P407	SGUK00000000	SAMN10908546	LAS
	YMC2017/09/C1287	RWYP00000000	SAMN10523658	JCVI
	YMC2017/12/C1072	RWYC00000000	SAMN10523670	JCVI
	YMC2017/12/R2228	RWXX00000000	SAMN10523665	JCVI
	YMC2018/09/B10953	SGUO00000000	SAMN10908814	LAS
	YMC2017/10/U3487	RWWI00000000	SAMN10523653	JCVI
	YMC2017/11/R2476	RWYF00000000	SAMN10523673	JCVI
	YMC2017/09/B12393	RWYO00000000	SAMN10523657	JCVI
	YMC2017/10/C794	RWWH00000000	SAMN10523652	JCVI
	YMC2017/11/U508	RWWF00000000	SAMN10523649	JCVI
	YMC2017/11/R1876	RWWE00000000	SAMN10523648	JCVI
	YMC2017/11/R2589	RWWD00000000	SAMN10523647	JCVI
K. pneumoniae	YMC2017/11/R3916	RWWC00000000	SAMN10523646	JCVI
	YMC2017/12/R1651	RWYA00000000	SAMN10523668	JCVI
	YMC2017/12/R337	RWXZ00000000	SAMN10523667	JCVI
	YMC2017/12/B1305	RWXY00000000	SAMN10523666	JCVI
	YMC2017/12/B2595	RWXT00000000	SAMN10523661	JCVI
	YMC2017/10/R1014	RWYN00000000	SAMN10523656	JCVI
	YMC2017/12/P169	RWWA00000000	SAMN10523644	JCVI
	YMC2017/12/U2812	RWXV00000000	SAMN10523663	JCVI
	YMC2017/12/B5771	RWXU00000000	SAMN10523662	JCVI
	YMC2017/12/P686	RWXS00000000	SAMN10523660	JCVI
	YMC2016/02/N279	SGUT00000000	SAMN10908872	LAS
	YMC2017/12/R3972	RWYQ00000000	SAMN10523659	JCVI
	YMC2017/12/P781	RWYE00000000	SAMN10523672	JCVI
	YMC2016/08/N460	SGUL00000000	SAMN10908808	LAS
	YMC2017/03/R348	RWXH00000000	SAMN10523624	JCVI
	YMC2017/01/B12075	RWVV00000000	SAMN10523639	JCVI
	YMC2017/02/B87	RWVU00000000	SAMN10523638	JCVI
	YMC2017/02/B4520	RWVQ00000000	SAMN10523634	JCVI

A. baumannii	YMC2017/02/B4039	RWXI00000000	SAMN10523625	JCVI
	YMC2017/02/R4043	RWXG00000000	SAMN10523623	JCVI
	YMC2017/03/B664	RWVR00000000	SAMN10523635	JCVI
	YMC2017/03/B5000	RWXJ00000000	SAMN10523626	JCVI
	YMC2017/03/R3279	RWVN00000000	SAMN10523631	JCVI
	YMC2017/04/R488	RWXE00000000	SAMN10523621	JCVI
	YMC2017/04/R640	RWVO00000000	SAMN10523632	JCVI
	YMC2017/05/B13743	RWXL00000000	SAMN10523628	JCVI
	YMC2017/06/B7857	RWXK00000000	SAMN10523627	JCVI
	YMC2017/06/B8443	RWVS00000000	SAMN10523636	JCVI
	YMC2017/06/B10945	RWVP00000000	SAMN10523633	JCVI
	YMC2017/07/B1155	RWVM00000000	SAMN10523630	JCVI
	YMC2014/07/R649	RWXF00000000	SAMN10523622	JCVI
	YMC2017/07/R832	RWXC00000000	SAMN10523619	JCVI
	YMC2017/07/R2000	RWXD00000000	SAMN10523620	JCVI
	YMC2017/07/R1800	RWVZ00000000	SAMN10523643	JCVI
	YMC2017/10/B943	RWVT00000000	SAMN10523637	JCVI
	YMC2016/11/B8245	RWVY00000000	SAMN10523642	JCVI
	YMC2016/11/B12450	RWVW00000000	SAMN10523640	JCVI
	YMC2017/07/R17	RWVL00000000	SAMN10523629	JCVI
	YMC2016/11/B9337	RWVX00000000	SAMN10523641	JCVI
P. aeruginosa	YMC2017/08/R997	RWWN00000000	SAMN10523603	JCVI
	YMC2017/08/R2483	RWXP00000000	SAMN10523596	JCVI
	YMC2017/08/R4730	RWWK00000000	SAMN10523600	JCVI
	YMC2017/08/R4164	RWWQ00000000	SAMN10523606	JCVI
	YMC2017/07/R3870	RWWO00000000	SAMN10523604	JCVI
	YMC2017/08/R4656	RWXM00000000	SAMN10523592	JCVI
	YMC2017/08/R4537	RWWR00000000	SAMN10523607	JCVI
	YMC2017/12/B1486	RWWV00000000	SAMN10523611	JCVI
	YMC2017/08/R2637	RWXN00000000	SAMN10523593	JCVI
	YMC2017/08/R3839	RWXQ00000000	SAMN10523597	JCVI
	YMC2017/08/R4671	RWWL00000000	SAMN10523601	JCVI
	YMC2017/08/R4369	RWXO00000000	SAMN10523594	JCVI
	YMC2017/08/R4244	RWWX00000000	SAMN10523613	JCVI
	YMC2017/08/R4393	RWWS00000000	SAMN10523608	JCVI
	YMC2017/08/R4643	RWWZ00000000	SAMN10523615	JCVI
	YMC2017/08/R4722	RWWT00000000	SAMN10523609	JCVI
	YMC2017/09/R486	RWWU00000000	SAMN10523610	JCVI
	YMC2017/08/R3890	RWXR00000000	SAMN10523598	JCVI
	YMC2017/08/R4261	RWWY00000000	SAMN10523614	JCVI
	YMC2017/08/B13455	RWXA00000000	SAMN10523616	JCVI
	YMC2017/09/R3207	RWXB00000000	SAMN10523617	JCVI
	YMC2017/09/B348	RWWW00000000	SAMN10523612	JCVI
	YMC2017/08/U4581	RXFP00000000	SAMN10523618	JCVI
	YMC2017/08/U1849	RWWP00000000	SAMN10523605	JCVI
	YMC2017/08/U3484	RWWM00000000	SAMN10523602	JCVI
	YMC2017/06/R4480	RWWJ00000000	SAMN10523599	JCVI
