# Supplementary figures and images for: In Vitro Activity of a Novel Siderophore-Cephalosporin LCB10-0200 (GT-1), and LCB10-0200/Avibactam, against Carbapenem-Resistant Escherichia coli, Klebsiella pneumoniae, Acinetobacter baumannii, and Pseudomonas aeruginosa Strains at a Tertiary Hospital in Korea
Source: Pharmaceuticals (Basel). 2021 Apr 16;14(4):370. doi: 10.3390/ph14040370 (PMC8072773; doi:10.3390/ph14040370)

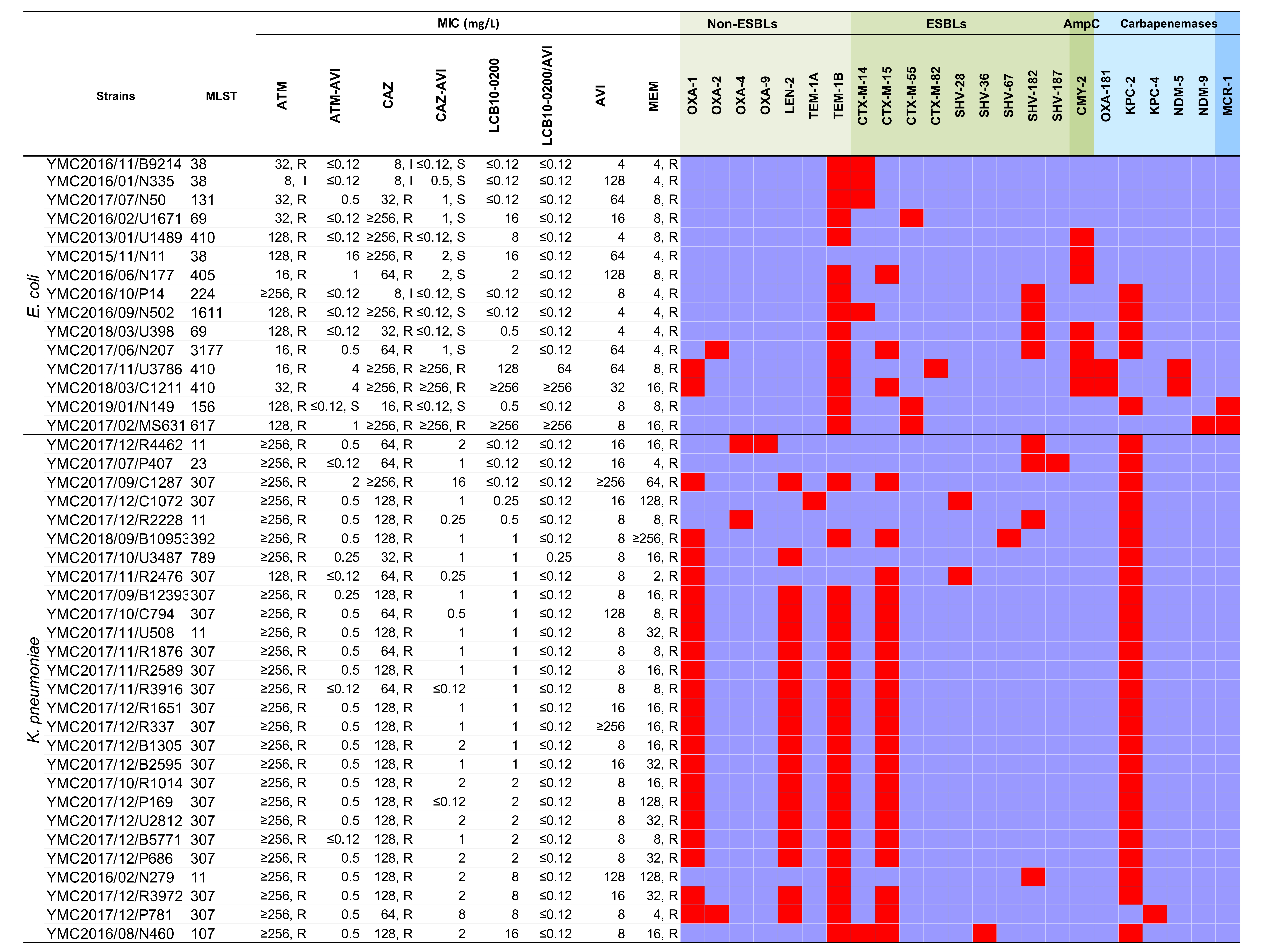

Supplement: Supplementary file 1 [file pharmaceuticals-14-00370-s001.zip › Figure 1. Antibiotic susceptibility and resistome of the carbapenem-resistant E. coli and K. pneumoniae strains.jpg]

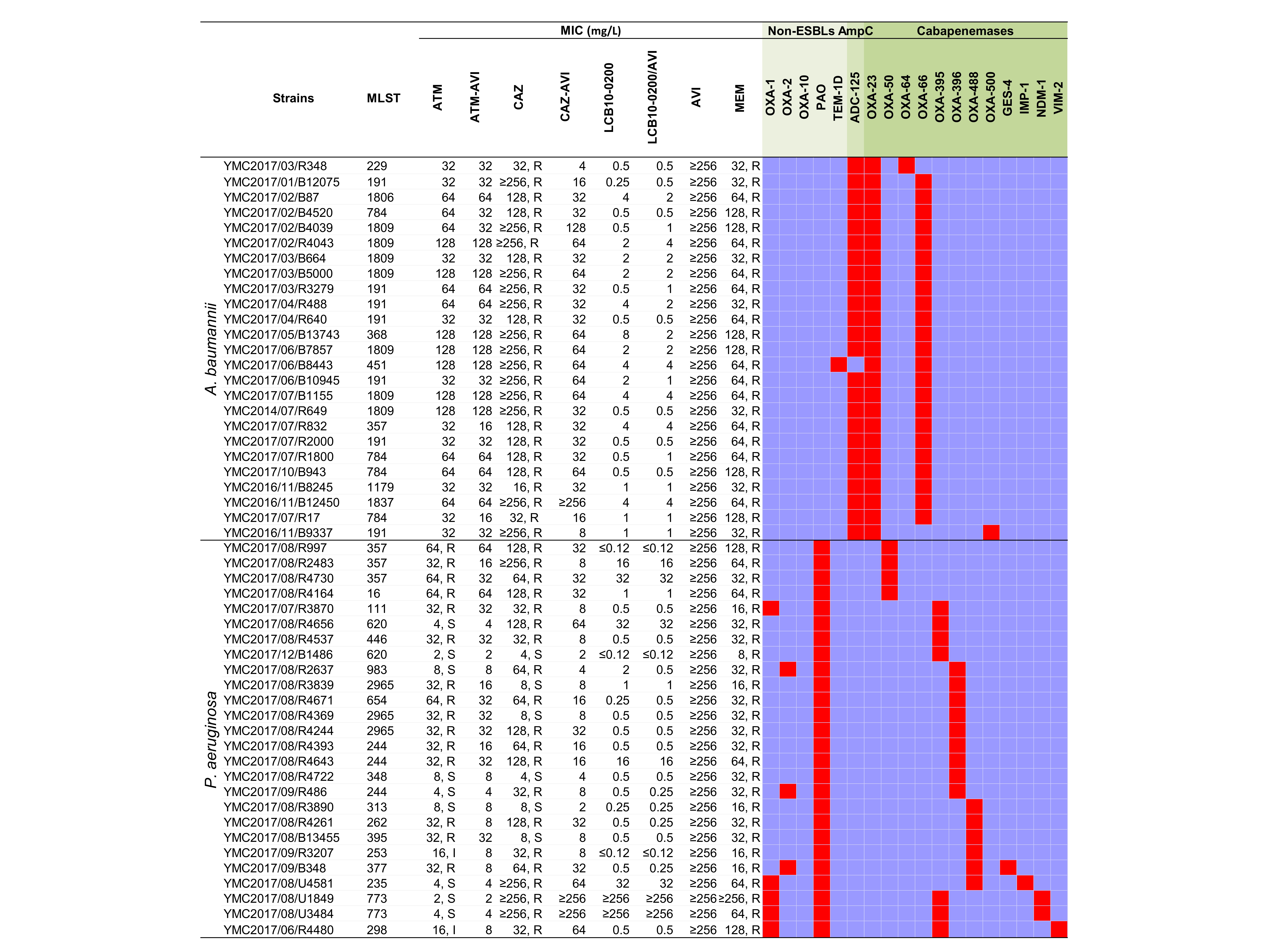

Supplement: Supplementary file 1 [file pharmaceuticals-14-00370-s001.zip › Figure 3.Antibiotic susceptibility and resistome of the carbapenem-resistant A. baumannii and P. aeruginosa strains.jpg]
